# The *Schizosaccharomyces pombe* JmjC-Protein, Msc1, Prevents H2A.Z Localization in Centromeric and Subtelomeric Chromatin Domains

**DOI:** 10.1371/journal.pgen.1000726

**Published:** 2009-11-13

**Authors:** Luke Buchanan, Mickaël Durand-Dubief, Assen Roguev, Cagri Sakalar, Brian Wilhelm, Annelie Strålfors, Anna Shevchenko, Rein Aasland, Andrej Shevchenko, Karl Ekwall, A. Francis Stewart

**Affiliations:** 1 Genomics, BioInnovationsZentrum, Technische Universität Dresden, Dresden, Germany; 2 Max Planck Institute of Molecular Cell Biology and Genetics, Dresden, Germany; 3 Karolinska Institute, Department of Biosciences and Medical Nutrition, NOVUM, Huddinge, Sweden; 4 Research Institute for Immunology and Cancer, University of Montreal, Montreal, Quebec, Canada; 5 Department of Molecular Biology, University of Bergen, Bergen, Norway; University of Cambridge, United Kingdom

## Abstract

Eukaryotic genomes are repetitively packaged into chromatin by nucleosomes, however they are regulated by the differences between nucleosomes, which establish various chromatin states. Local chromatin cues direct the inheritance and propagation of chromatin status via self-reinforcing epigenetic mechanisms. Replication-independent histone exchange could potentially perturb chromatin status if histone exchange chaperones, such as Swr1C, loaded histone variants into wrong sites. Here we show that in *Schizosaccharomyces pombe*, like *Saccharomyces cerevisiae*, Swr1C is required for loading H2A.Z into specific sites, including the promoters of lowly expressed genes. However *S. pombe* Swr1C has an extra subunit, Msc1, which is a JumonjiC-domain protein of the Lid/Jarid1 family. Deletion of Msc1 did not disrupt the *S. pombe* Swr1C or its ability to bind and load H2A.Z into euchromatin, however H2A.Z was ectopically found in the inner centromere and in subtelomeric chromatin. Normally this subtelomeric region not only lacks H2A.Z but also shows uniformly lower levels of H3K4me2, H4K5, and K12 acetylation than euchromatin and disproportionately contains the most lowly expressed genes during vegetative growth, including many meiotic-specific genes. Genes within and adjacent to subtelomeric chromatin become overexpressed in the absence of either Msc1, Swr1, or paradoxically H2A.Z itself. We also show that H2A.Z is N-terminally acetylated before, and lysine acetylated after, loading into chromatin and that it physically associates with the Nap1 histone chaperone. However, we find a negative correlation between the genomic distributions of H2A.Z and Nap1/Hrp1/Hrp3, suggesting that the Nap1 chaperones remove H2A.Z from chromatin. These data describe H2A.Z action in *S. pombe* and identify a new mode of chromatin surveillance and maintenance based on negative regulation of histone variant misincorporation.

## Author Summary

Chromatin is based on a repetitive structural unit called the nucleosome. However, the regulatory properties of chromatin are mediated by the differences between nucleosomes, due to post-translational modifications or the inclusion of histone variants. These differences are maintained by inheritance through *cis*-acting epigenetic mechanisms. Here we describe a case where the local character of chromatin is not only determined by *cis*-acting mechanisms but also negatively regulated in trans. The case involves loading of the histone H2A variant, H2A.Z, into chromatin. We show that H2A.Z in the yeast *Schizosaccharomyces pombe* is mainly found in genes at the first transcribed nucleosome and is inserted into this nucleosome by the Swr1C remodeling machine. However, Swr1C has a regulatory subunit, Msc1, which is not required for H2A.Z promoter loading but prevents H2A.Z occupancy in the inner centromere and subtelomeric regions. These two specialized regions are neither eu- nor heterochromatin and share certain characteristics, which may predispose them to the aberrant inclusion of H2A.Z and the requirement for trans regulation by Msc1.

## Introduction

Chromatin is based on a repetitive structural unit called the nucleosome. However the regulatory properties of chromatin are mediated by the differences between nucleosomes, due to post-translational modifications or presence of histone variants. Cytologically, chromatin was initially divided into heterochromatin and euchromatin [1]. The underlying molecular basis of this division was established at the nucleosomal level after the discovery of the partitioning of histone lysine methylations into hetero- and euchromatic domains [2],[3],[4]. Further degrees of chromatin specificity have been revealed by studies of histone modifications and variants. For example, trimethylation of histone 3 at lysine 4 (H3K4me3) characterizes nucleosomes around RNAP II promoters [5] while incorporation of the histone 3 variant CENP-A characterizes nucleosomes of the inner centromere [6]. How these differences arise and propagate, often at individual nucleosomes, is not clear, although clues are available. For example, self-reinforcing feed-forward mechanisms can explain the propagation of nucleosomal states [7],[8],[9]. These mechanisms rely upon a physical connection between a protein that binds a histone modification with an enzyme that catalyzes the same modification. Notable examples include the association between H3K9 methyltransferase Clr4 and H3K9 methylation [10], and Spp1 and Set1 for H3K4 methylation [11].

Another way to maintain nucleosomal differences and chromatin domains are boundary mechanisms. By blocking the spread of a self-reinforcing mechanism, boundaries such as those provided by insulators [12] or TFIIIC binding sites [13] restrict chromatin states to their respective domains. Boundaries based on DNA cis elements are pre-fixed. Other boundaries can be variably positioned depending upon expression levels of position effect variegation proteins, which enhance or diminish the spread of heterochromatin [14]. However most explanations of self-reinforcing mechanisms and boundary phenomena assume that chromatin is one-dimensional. Because it is obviously three-dimensional and apparently confined within a single cellular compartment, what mechanisms prevent the chaotic distribution of nucleosomal identities?

This question is especially relevant for the processes that exchange canonical histones for histone variants. After DNA replication, both daughter DNA molecules must be packaged in the same chromatin status as the parental molecule. Canonical histone deposition occurs in a replication-coupled (RC) manner. However, the deposition of certain histone variants occurs in a DNA replication-independent (RI) manner [15]–[18]. For example, the H3 variant H3.3 is targeted to chromatin via an RI transcription-coupled mechanism [19],[20] involving the H3.3-specific chaperone HIRA, as opposed to the RC chaperone CAF1, which incorporates H3.1 [21].

RI chaperones are particularly susceptible to mistargeting of histone variants. For example, the H3 variant CENP-A is enriched in the centromeric domain under guidance from neighbouring heterochromatin and epigenetic mechanisms [22],[23]. The histone chaperone RbAp48 interacts with CENP-A and is required for CENP-A loading. However, RbAp48 interacts with both the CAF1 and HIRA chaperone complexes and can load either CENP-A or canonical H3 into chromatin *in vitro* [24],[25]. Furthermore, overexpression of CENP-A in various organisms leads to aberrant deposition in euchromatin [26],[27], and defects in CAF1 or HIRA nucleosome assembly pathways also lead to mistargeting of budding yeast CENP-A (Cse4) [28].

In this paper we focus on the H2A variant, H2A.Z, which is incorporated into budding yeast chromatin by Swr1, the catalytic subunit of the Swr1 complex (Swr1C) and one of the SWI2/SNF2 superfamily of ATPase chromatin remodelers [29]–[33]. Swr1C deposits H2A.Z-H2B dimers in chromatin both *in vitro* and *in vivo*, but does not remove H2A.Z from chromatin [32].

In budding yeast, H2A.Z is mainly positioned at the promoters of lowly expressed or inducible genes [34]–[38] and is lost upon gene induction [30],[31]. At least some of these promoters show reduced induction in the absence of H2A.Z, suggesting that the destabilization of promoter nucleosomes by the inclusion of H2A.Z facilitates transcriptional initiation [35]. H2A.Z may also be involved in defining chromatin boundaries and domains. The absence of H2A.Z, or NuA4-mediated H2A.Z acetylation, allows telomeric gene silencing to spread beyond its usual domain resulting in the repression of sub-telomeric gene expression [39],[40].

H2A.Z has also been implicated in centromere function and chromosome segregation in mammals [41], budding yeast [29],[42] and fission yeast [43],[44], evident in increased rates of chromosomal loss in H2A.Z mutants and genetic interactions between H2A.Z and microtubule components [45]. H2A.Z localizes to centric and pericentric chromatin in mammals [46] but was not found at centromeres in budding yeast [35].

Hence H2A.Z and Swr1C are involved in many aspects of chromatin regulation. Central to these processes is the incorporation of H2A.Z into specific nucleosomes. However the basis for this specificity is unclear. Here we report that this process is due to both positive and negative target selectivity by Swr1C, due in part to the JmjC-domain protein, Msc1, which is a stoichiometric subunit of the fission yeast Swr1C. Msc1 negatively regulates H2A.Z incorporation into specific chromatin regions at the inner centromere and sub-telomere.

## Results

### The fission yeast Swr1 complex contains Msc1 as a stoichiometric subunit

**Figure 1:**
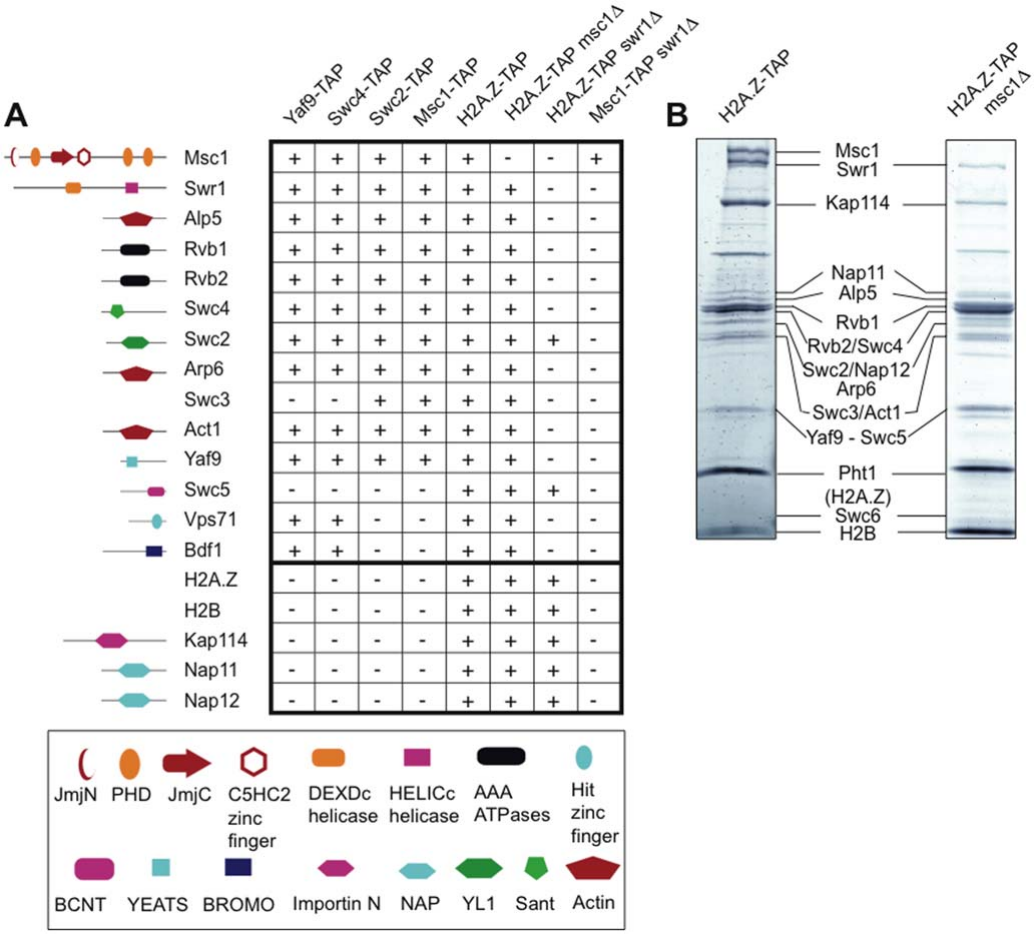
Msc1 is a stoichiometric subunit of the fission yeast Swr1C. (A) Swr1C subunits identified by LC-MS/MS after TAP-tag purification from multiple baits, as indicated. Protein domains identified in Swr1C subunits by SMART (EMBL-EBI) are depicted to the left. (B) Coomassie stained PAGE gels of H2A.Z-TAP and H2A.Z-TAP/*msc1*Δ purifications.

As part of a study to develop datasets for comparative proteomics, we purified a *S. pombe* complex with high subunit orthology to the *S. cerevisiae* Swr1 complex [47]. To characterize this complex in greater detail, we applied a sequential tagging strategy [48] to purify Swr1C via its Yaf9, Swc4, Swc2 and Msc1 subunits, as well as via Pht1 (which is the fission yeast histone variant H2A.Z). Notably, each of the tagged proteins, with the exception of Pht1, appeared to be stoichiometric Swr1C subunits with no indication that any of them exist as free protein in the cell or as part of another complex (Figure 1B and data not shown).

Msc1 is a JmjC domain protein, which has no orthologue in the *S. cerevisiae* Swr1C. Msc1 is a member of the highly conserved Lid/Jarid1 family and has five zinc fingers, including one JmjN and three PHD fingers, an ARID/BRIGHT AT rich DNA binding domain and a Plu domain (Figure S1). Msc1 was initially identified as a multi-copy suppressor of the absence of the cell cycle progression kinase, Chk1 [49], and has been recently linked with H2A.Z action [43].

To investigate the role of Msc1 in Swr1C complex integrity, immunoprecipitations were performed using H2A.Z-TAP in an *msc1*Δ strain. All Swr1C subunits except Msc1 were detected. Therefore Msc1 is not required for the association of any other subunit or the association of Swr1C with H2A.Z (Figure 1A and 1B). Swr1 itself is essential for complex integrity, demonstrated by the absence of most Swr1C members in H2A.Z-TAP/*swr1*Δ and Msc1-TAP/*swr1*Δ purifications. Notably the association of Swc2 and Swc5 in the H2A.Z-TAP/*swr1*Δ experiment indicates that these subunits directly bind H2A.Z. Msc1 appears to be a stoichiometric subunit of Swr1C based on the intensity of its band in Coomassie stained PAGE gels, its presence in immunoprecipitations from multiple Swr1C baits and the ability of Msc1-TAP to pull down a complete Swr1C.

In addition to Swr1C, the H2A.Z-TAP purifications also yielded H2B, the Nap1/Nap1.2 histone chaperones, and the importin family protein Kap114 (Figure 1). These proteins were not detected from Yaf9-, Swc4-, Swc2-, Msc1- or Swr1-TAP purifications but were detected in H2A.Z-TAP/*swr1*Δ, demonstrating they are H2A.Z-specific and do not interact directly with Swr1C but only with H2A.Z itself. A similar interaction between H2A.Z and Nap1 in *S. cerevisiae* has been reported [30],[32].

### Loss of Msc1 leads to less H2A.Z in euchromatin

**Figure 2:**
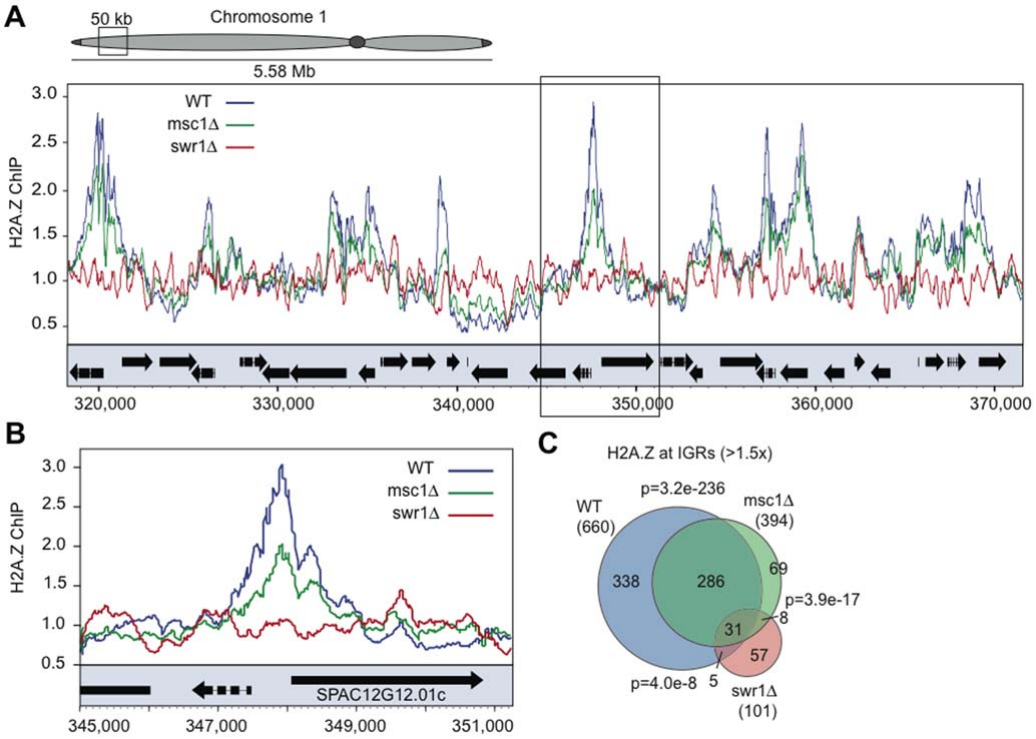
H2A.Z deposition in euchromatin requires Swr1 but not Msc1. (A) A representative 50kb region of euchromatin on chromosome 1 is shown displaying H2A.Z-myc ChIP enrichment over unprecipitated chromatin input as measured by ChIP-chip. H2A.Z-myc enrichment is shown in WT (blue), *msc1*Δ (green), and *swr1*Δ (red). Open reading frames (ORFs) are represented by black boxes (arrows indicate ORF direction) below the ChIP-chip traces. (B) A region from the inset in (A) containing *SPAC12G12.01c* is shown at higher resolution. This peak was validated by conventional ChIP (Figure S2). C) IGRs enriched in H2A.Z (using a >1.5× cutoff) in each of the three strains were plotted as a Venn diagram to illustrate the overlap between the data sets. P values were generated using the hypergeometric method, and represent the probability of producing the given overlap between each pair of the shown datasets.

To investigate the role(s) of Msc1 in H2A.Z metabolism, we performed genome-wide chromatin immunoprecipitation (ChIP-chip) analyses using myc-tagged H2A.Z in WT, *msc1*Δ and *swr1*Δ strains. As shown for a representative euchromatic region (Figure 2A), H2A.Z peaks were found predominantly at promoters in WT but were absent in *swr1*Δ strains. In the absence of Msc1, these peaks were found in the same places but often diminished.

To assess the genome-wide distribution of H2A.Z statistically, the tiling array data for every gene was represented by two values corresponding to the upstream intergenic region (IGR) and the open reading frame (ORF). At a cutoff of >1.5×, 660 IGRs showed enrichment for H2A.Z, indicating that about 1/7^th^ of promoters in vegetative, exponentially growing, *S. pombe* contain strongly enriched H2A.Z (Figure 2C). This is a very similar value to *S. cerevisiae* [34],[35]. Furthermore only about 40% of all H2A.Z promoter peaks remained above the 1.5× threshold in the absence of Msc1 (Figure 2C).

**Figure 3:**
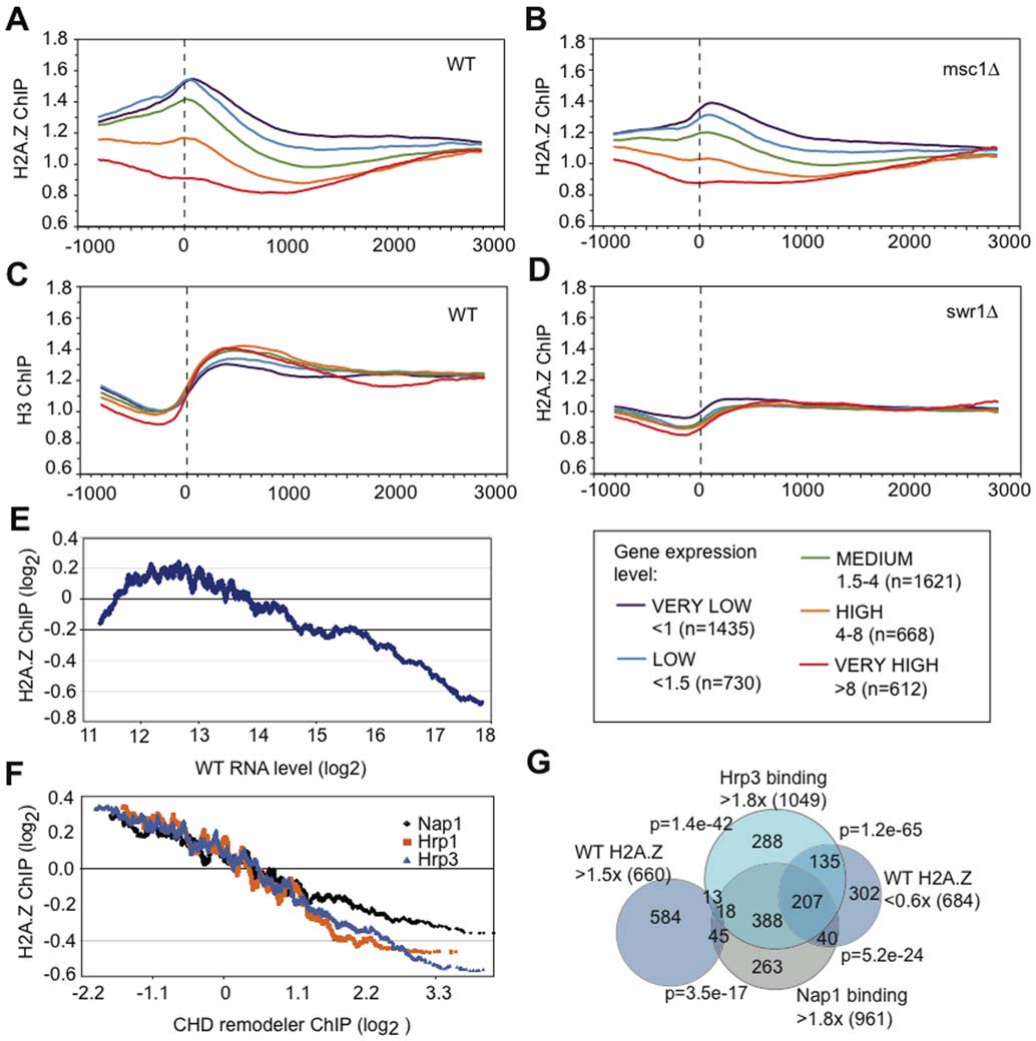
H2A.Z peaks inversely correlate with expression level and Nap/Hrp occupancy. (A–D) All data from the 20bp tiling arrays were ordered with respect to the initiating methionine of each gene, which is shown as zero and the dotted line. The data were then binned into five groups according to the expression level of the gene [52] as indicated in the box at the right. (A) H2A.Z-myc ChIP from WT. (B) H2A.Z ChIP from *msc1*Δ. (C) H3 ChIP from WT. (D) H2A.Z-myc ChIP from *swr1*Δ. E) IGRs enriched in H2A.Z inversely correlate with mRNA expression level except for the least expressed 5%. H2A.Z enrichment (log2) was plotted against absolute mRNA level (log2) in WT using a 150-gene moving average based on expression data from Wiren et al, 2005 [52]. F) Moving average plots of H2A.Z enrichment against Hrp1, Hrp3 and Nap1 binding at IGRs using ChIP data from Walfridsson et al, 2007 [51]. Genes were sorted according to increasing Hrp1, Hrp3, or Nap1 IGR enrichment, and moving average plots of Log2 H2A.Z IGR values were generated using a 150-gene moving average. G) Venn diagram that represents the overlap of IGRs either enriched (>1.5) or depleted (,0.6) in H2A.Z, with IGRs enriched in either Hrp3 or Nap1 (.1.8). P values were generated using the hypergeometric method, and represent the probability of producing the given overlap between each pair of the shown datasets, except for the two values related to WT H2A.Z >1.5×, which refer to the lack of overlap.

We further divided the occurrence of H2A.Z peaks into five categories with respect to mRNA expression level from very low to very high (Figure 3A–3D). In WT the H2A.Z peak corresponds with the first nucleosome in the transcribed region (Figure 3A) and the nucleosome-sparse promoter region can be seen as the low point in the H3 ChIP at −200 (Figure 3C). The H2A.Z peak does not correspond to the region of peak H3 density, which is found at +300 and presumably reflects peak nucleosomal density. Furthermore, the most lowly expressed genes have higher H2A.Z peaks and the most highly expressed genes do not appear to have any H2A.Z at their promoters or elsewhere. Loss of Swr1 abolishes the H2A.Z peak as expected (Figure 3D), whereas loss of Msc1 results in a shift in all categories towards less H2A.Z, although the peak position remains the same (Figure 3B). Also notable is the absence of an H2A.Z peak at the −1 nucleosome, which is prominent in *S. cerevisiae* [5] but not *Drosophila* [50].

We further evaluated the relationship between H2A.Z promoter peaks and gene expression levels to observe a strong negative correlation (Figure 3E). As expected from Figure 3A, H2A.Z occupancy inversely correlates with mRNA abundance. However this inverse correlation does not apply to the least expressed genes.

Furthermore we observed strong positive correlations between H2A.Z peaks and H4K16, H3K14 and other histone tail acetylations (Figure S2). These proteomic and ChIP-chip data confirm that Swr1 and Swr1C are required for loading of H2A.Z into promoter sites in *S. pombe* euchromatin, whereas the role(s) for Msc1 are more subtle. Msc1 is not required to specify the sites of H2A.Z loading, rather it contributes to H2A.Z occupancy either through loading efficiency or persistence.

### H2A.Z is depleted at IGRs bound by Nap1 and CHD chromatin remodelers

Notably we also observed a strong inverse correlation between the genome wide distributions of H2A.Z and the nucleosome chaperones, Nap1, Hrp1 and 3 [51],[52]. Moving average plots show H2A.Z enrichment decreases with increasing Hrp1, Hrp3 and Nap1 binding at IGRs across the genome (Figure 3F). IGRs bound by H2A.Z (>1.5× cut-off) do not coincide with IGRs bound by Nap1 and Hrp1 (>1.8× cut-off). In contrast, IGRs depleted in H2A.Z (<0.60×) show a strong overlap with IGRs bound by Hrp1 and Nap1 (Figure 3G). Considering that Nap1 physically interacts with H2A.Z, but not Swr1C, the absence of H2A.Z at Nap1-, Hrp1- and Hrp3-bound intergenic chromatin suggests that H2A.Z is removed from chromatin by Nap1 and the CHD remodelers.

### Msc1 negatively regulates H2A.Z inclusion at centromeres

**Figure 4:**
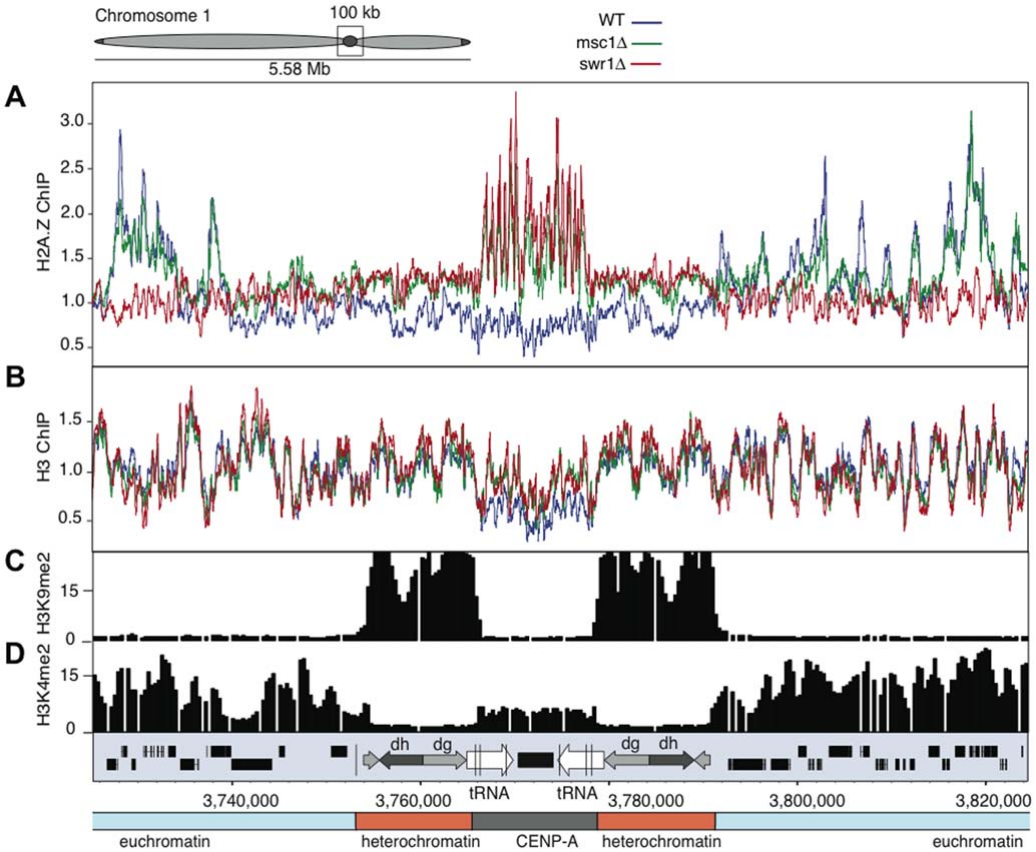
H2A.Z is incorporated in the inner centromeres in *msc1*Δ and *swr1*Δ. (A) ChIP-chip binding profiles for H2A.Z-myc, (B) H3, (C) H3K9me2, and (D) H3K4me2 at the centromere and flanking euchromatin regions of chromosome 1. Data for (C) and (D) from Cam et al, 2005 [53]. Open reading frames and repeat elements present at the centromeric region are indicated below.

Normally H2A.Z is absent from all centromeric regions, including both the CENP-A containing inner centromere and the pericentric heterochromatin (Figure 4A). However, in the absence of Msc1 or Swr1, H2A.Z became incorporated specifically in the inner centromere (Figure 4A, Figure S3). This corresponded to increased H3 (Figure 4B) and decreased CENP-A [43] occupancy. Centromeric H2A.Z in the *msc1*Δ strain demonstrates that Msc1 acts as a negative regulator of H2A.Z inclusion or persistence at centromeres. Additionally, the presence of centromeric H2A.Z in *swr1*Δ implies that H2A.Z does not strictly rely on Swr1C for loading into chromatin.

### Msc1 is required for sub-telomeric chromatin state maintenance

**Figure 5:**
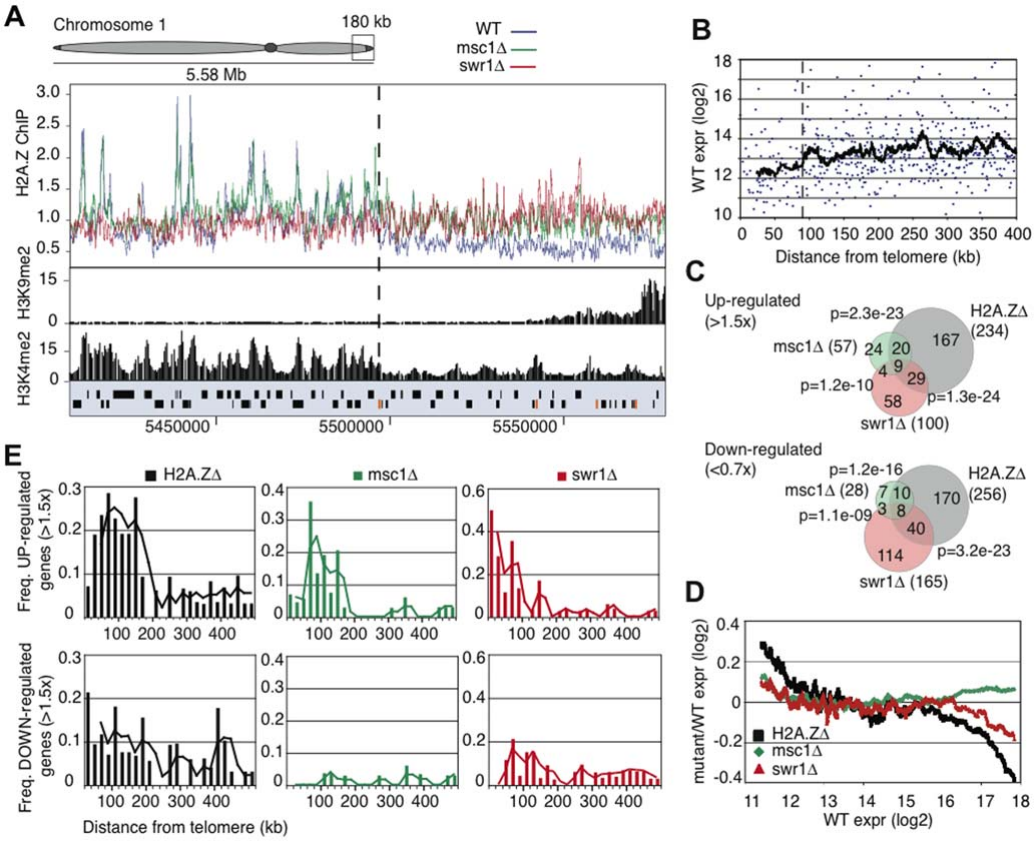
Msc1 is required for gene silencing in sub-telomeres. (A) ChIP-chip binding profiles for H2A.Z-myc, H3K9me2, and H3K4me2 [53] for 180kb at the end of chromosome 1. Open reading frames are represented by black boxes and LTR retrotransposon elements by orange boxes. The dotted lines indicates the transition point between eu- and ST-chromatin domains. (B) Absolute RNA level in WT (log2) plotted against distance from nearest telomere for all genes in the fission yeast genome within 400 kb of telomeres on chromosomes 1 and 2 using data from Wiren et al, 2005 [52]. The black line represents a 20 gene moving average. The dotted line represents a transition point in gene expression approximately 90 kb from telomere ends. (C) Venn diagrams represent the number of genes either up (>1.5×) or down (<0.7×) regulated in *H2A.Z*Δ, *msc1*Δ or *swr1*Δ and the overlap between the three datasets. (D) Changes in RNA level over WT (log2 ratio) in each of the three mutants (*H2A.Z*Δ, *msc1*Δ or *swr1*Δ) were plotted as a moving average against WT RNA level. (E) Changes in RNA level over WT (log2 ratio) were plotted against genomic distance from the nearest telomere. Genes were grouped into 20 kb windows and the frequency of up- and down-regulated genes calculated for each 20 kb window.

Similar to the centromeres, H2A.Z deposition at sub-telomeric domains was also affected by the losses of Msc1 and Swr1. In WT, H2A.Z is depleted from sub-telomeric domains (approximately 100 kb in size) at the left and right ends of chromosomes 1 and 2 (Figure 5A, Figure S4). Loss of either Msc1 or Swr1 caused an increase of H2A.Z in these sub-telomeric domains. The increase of H2A.Z was not as dramatic as that observed at centromeres and H2A.Z distribution did not adopt the euchromatic pattern of IGR promoter peaks, rather it was more scattered. Notably the transition between euchromatin and the H2A.Z-free subtelomeric chromatin appears to be quite sharp on all four chromosome ends (Figure 5A; Figure S4). The subtelomeric regions of chromosome 3 do not show H2A.Z depletion, or increased enrichment in the mutants, most likely because the rRNA gene repeats occupy both ends of this chromosome (Figure S4).

The sharp transition between euchromatin and sub-telomeric chromatin also corresponds to a transition of H3K4me2 levels [53]. Notably this sharp transition coincides with the presence of LTR elements in at least two of the four cases (Figure 5A, Figure S4). Genes residing in these sub-telomeric regions also tend to be the most lowly expressed [52], [54]–[57] with an apparently sharp boundary corresponding to H3K4me2 and H2A.Z transitions (Figure 5B). Furthermore, at least for one subtelomeric region, Swi6 binding, which spreads from the densely H3K9 methylated telomeric region, appears to reach the same boundary [58]. Based on these observations, we propose that the subtelomeric regions represent a distinct class of chromatin, and suggest the term ST-chromatin, which has different regional properties than bulk eu- or heterochromatin. Examination of our genome-wide ChIP-chip datasets [52] further revealed that ST-chromatin is also depleted in H4K5, H4K12, H4K16 and H3K14 acetylation, and has a higher H3 density. These regions are highly enriched for genes that are upregulated during meiosis, stress, and after the loss of Clr3 or Hrp1/Hrp3 (Table 1).

**Table 1:** Histone and gene expression characteristics of ST-(subtelomeric) chromatin showing all correlations displaying a P value below E-02 using data from [51]–[53], [54]–[57], this paper and unpublished data.

Histones	IGR or ORF	Fold vs av. WT genome	Overlapping (of 140)	P value	Ref
H2A.Z	IGR	<0.6	36	2.0E-12	here
	ORF	<0.7	39	4.7E-06	here
H3	ORF	>1.2	21	8.1E-04	52
H3K4me2	IGR	<1.5	57	1.1E-05	53
	ORF	<1.5	63	9.9E-07	53
H3K9Ac	IGR	<2.0	40	6.6E-03	52
H3K14Ac	IGR	<1.5	49	1.8E-04	52
H4K5Ac	IGR	<1.5	58	5.5E-07	52
	ORF	<1.5	65	2.4E-08	52
H4K12Ac	IGR	<1.5	58	2.0E-07	52
	ORF	<1.5	59	1.4E-06	52
H4K16Ac	IGR	<1.5	56	7.2E-04	52
**Gene expression**					
Meiosis	ORF	>2.0	66	3.9E-06	54–57
Stress	ORF	>2.0	49	2.0E-06	54–57
clr3Δ	ORF	>1.5	32	1.9E-16	52
hrp1Δ/hrp3Δ	ORF	>2.0	28	3.1E-10	51
clr4Δ	ORF	>1.5	10	1.5E-03	55

Like ST-chromatin, the inner centromeric (IC) domain is depleted in H3K4me2 compared to levels typically found in euchromatin [53], (see Figure 4D and Figure 5A). Hence H3K4me2 and H2A.Z are similarly depleted at WT sub-telomeres and inner centromeres, and both chromatin domains display increased H2A.Z enrichment in *swr1*Δ and *msc1*Δ strains.

### Msc1 is required for sub-telomeric gene silencing

Gene expression changes in the absence of Msc1, Swr1 and H2A.Z were measured by microarray analysis. A significant overlap between the three datasets was found (Figure 5C) demonstrating that a common set of genes is affected in all three mutants. In *msc1*Δ, very few genes were misregulated (either up >1.5×, or down <0.67×; 85) compared to *swr1*Δ (265) or *H2A.Z*Δ (490). Genes up-regulated in all three mutant strains were lowly expressed in WT. However loss of Msc1 had virtually no effect on the expression of any other genes, whereas loss of either H2A.Z or Swr1 also affected highly expressed genes (Figure 5D).

The most striking observation from the expression profiling was increased expression in the mutant strains of many genes within approximately 160kb of the ends of chromosomes 1 and 2 (Figure 5E). *H2A.Z*Δ, *msc1*Δ and *swr1*Δ strains all showed significant up-regulation of sub-telomeric genes, despite having either complete loss (*H2A.Z*Δ) or increased sub-telomeric deposition (*msc1*Δ and *swr1*Δ) of H2A.Z. The overlap between up-regulated genes in *H2A.Z*Δ, *msc1*Δ and *swr1*Δ was also higher at sub-telomeres than in the rest of the genome, indicating a similar loss of sub-telomeric transcriptional control in the three deletion strains (data not shown). In fact, more than 2/3rds of genes up-regulated in the absence of Msc1 lie in the sub-telomeric regions. Notably, up-regulation spreads beyond the ST-chromatin boundaries, suggesting that the loss of ST-chromatin and its boundaries caused neighbouring effects.

### Lysine acetylation of H2A.Z requires Swr1

**Figure 6:**
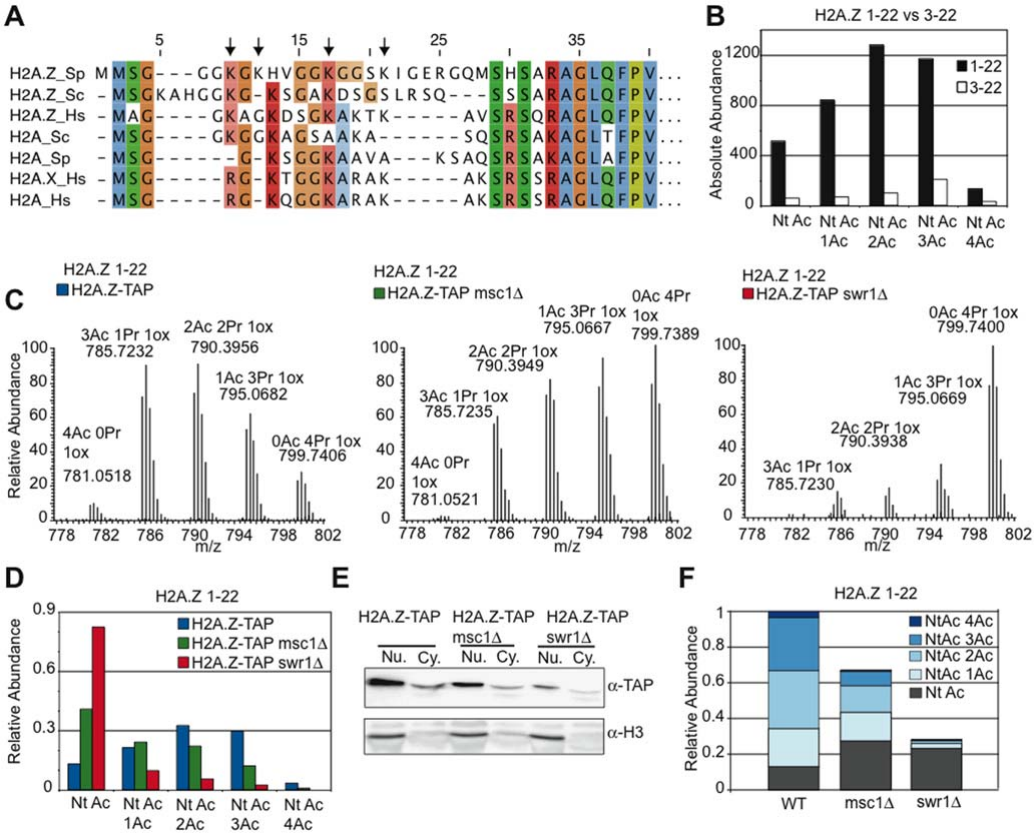
Lysine acetylation of H2A.Z requires Swr1 but not Msc1. (A) An alignment of N-terminal regions of H2A.Z and canonical H2A amino acid sequences from fission yeast (Sp), budding yeast (Sc) and human (Hs). (B) A comparison of absolute quantities of H2A.Z 1–22 and 3–22 isoforms as extracted from chromatogram peak areas. (C) MS spectra of H2A.Z 1–22 acetylation isoforms after propionyl anhydride treatment in WT, *msc1*Δ, and *swr1*Δ strains. Note that acetylation isoform peaks with fewer acetyl marks are greater in mass due to the propionyl (Pr) conversion of un-acetylated lysines (Pr causes 14Da greater mass than Ac). Due to the N-terminal methionines of H2A.Z, oxidation is common and can occur on either or both of the N-terminal methionines. Depicted are the spectra for 1-oxidation (1ox) isoforms of H2A.Z 1–22, the most abundant isoform (data not shown). (D) Relative quantification of the acetylation isoforms (using summed quantities of 0-, 1-, and 2-ox isoforms) of H2A.Z peptide 1–22 in WT, *msc1*Δ, and *swr1*Δ, demonstrating a strong reduction in acetylation in *swr1*Δ. (E) H2A.Z-TAP and H3 levels by Western blot of nuclear and cytoplasmic extracts. (F) Data combined from (D) and (E) to show absolute levels of H2A.Z and the N-terminal and lysine acetylations in WT, *msc1*Δ, and *swr1*Δ strains.

As a further way to evaluate H2A.Z biology and Msc1 action, we developed quantitative mass spectrometry for fission yeast histone post-translational modifications including the histone variant, H2A.Z. TAP-tagged H2A.Z was purified from WT, *msc1*Δ or *swr1*Δ strains with concomitant retrieval of associated H2B (Figure S5). Unexpectedly, we found that the *S. pombe* H2A.Z N-terminal amino acid sequence was incorrect because the genome sequence was wrongly edited (it has now been corrected). The correct sequence is presented in Figure 6A with a comparison to other H2As. H2A.Z has an extended N-terminal tail containing more lysines than canonical H2A. Also, *S. pombe* H2A.Z includes two N-terminal methionines, which are either both present or absent, resulting in two variations of the N-terminal peptide (named 1–22 or 3–22 respectively). A comparison of absolute levels of peptides 1–22 and 3–22 revealed that 1–22 is the major isoform. This isoform is always N-terminally acetylated. About 2/3rds of total H2A.Z also carries 2 or more lysine acetylations (Figure 6B and 6C, Figure S6). Hence the H2A.Z N-terminal tail is usually highly acetylated.

Multiple H2A.Z acetylation was reduced in *msc1*Δ and virtually abolished in *swr1*Δ strains (Figure 6C and 6D). Similarly, total H2A.Z levels were reduced by about 1/3^rd^ in *msc1*Δ and about 4-fold in *swr1*Δ strains (Figure 6E). We combined Figure 6D and 6E to estimate the abundance of acetylated forms in WT, *msc1*Δ and *swr1*Δ strains (Figure 6F). Notably, the absolute amount of H2A.Z that was acetylated only on the N-terminus increased in both mutant strains, whereas all species of lysine acetylations were decreased. In particular, lysine-acetylated H2A.Z almost vanished in the absence of Swr1, whereas the level of N-terminal-only acetylated H2A.Z increased. This near complete absence of multiply-acetylated H2A.Z coincides with the near complete absence of H2A.Z loading into chromatin in the absence of Swr1. Similarly, in the absence of Msc1 the reduction of multiply acetylated H2A.Z coincides with reduced H2A.Z occupancy, being approximately half in both cases. This suggests that multiple acetylation of H2A.Z requires incorporation into nucleosomes and that there is a pool of unincorporated nuclear H2A.Z which is not multiply acetylated. It also suggests that H2A.Z incorporated into nucleosomes in the absence of Msc1 is normally acetylated.

## Discussion

This work arose from our finding that a member of the highly conserved Lid/Jarid1 family, Msc1, is a subunit of the fission yeast Swr1C H2A.Z chaperone [47]. Here we show that Msc1 is not required for Swr1C integrity or binding of H2A.Z, however it is a stoichiometric subunit of the complex. Furthermore we found that the entire Swr1C can be biochemically purified using tagged H2A.Z, which also retrieves the Nap1 subunits of the CHD nucleosomal remodeler. To understand Msc1 function, we characterized H2A.Z metabolism in *S. pombe*.

### H2A.Z in *S. pombe*

Like in budding yeast, H2A.Z incorporation into euchromatin in *S. pombe* depends on Swr1C and tends to be found at promoters of lowly expressed genes. Apart from the most lowly expressed genes in vegetative growth, which are disproportionately found in subtelomeric regions [52], [55]–[58], there is a strong negative correlation between H2A.Z occupancy and mRNA expression level. There is also a strong negative correlation between H2A.Z and Nap1/Hrp1/Hrp3 CHD remodeler occupancy [51]. Because Nap1 binds to H2A.Z, we suggest that H2A.Z is loaded into many promoters and is removed by the CHD remodeler when the gene is expressed. Hence we suggest that the observed H2A.Z distribution in a ChIP experiment is like a ‘snap-shot’ of expression levels and only partially reflective of the sites into which H2A.Z was loaded. We propose that H2A.Z is loaded by Swr1C into the +1 nucleosome at most promoters and is subsequently removed by the Nap1/CHD remodeler upon transcription. This suggestion concords with similar suggestions for budding yeast [34],[35] and recent measurements of nucleosomal turnover, which occurs more rapidly at promoters [59]. In euchromatin, loss of Msc1 had a quantitative but not qualitative effect on H2A.Z promoter occupancy. It therefore appears that Msc1 does not play a role in defining the sites of H2A.Z deposition in euchromatin rather may contribute to the efficiency of reloading after Nap1/CHD removal in a transcription cycle.

**Figure 7:**
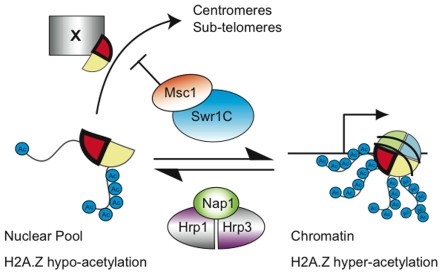
Regulatory cycle model for H2A.Z in *S. pombe*. Before loading into chromatin by Swr1C, the H2A.Z/H2B dimer is fully acetylated on the H2B tail but only N-terminally acetylated on the H2A.Z tail. After deposition in chromatin, mainly at the first transcribed nucleosome, H2A.Z becomes lysine acetylated. It may be removed from chromatin by the Nap1/CHD remodellers. Msc1 is required to negatively regulate H2A.Z inclusion into inner centromeric and subtelomeric chromatin by an unidentified mechanism (X). H2A.Z is represented by a red wedge, H2B by a yellow wedge.

By quantitative mass spectrometry, we found that H2A.Z is always N-terminally acetylated but variably acetylated on four lysines in the N-terminal tail. In the absence of Swr1, very little H2A.Z was found in chromatin and very little became multiply acetylated. Furthermore, the N-terminally acetylated form of H2A.Z persisted regardless of the absence of Swr1 but overall H2A.Z levels were reduced, which equated with the absence of the multiply acetylated forms. In agreement with similar suggestions from work with *S. cerevisiae* [39],[60],[61], we conclude that lysine acetylation of H2A.Z depends upon loading into chromatin. Notably, H2B associated with H2A.Z was heavily acetylated regardless of whether it was loaded into chromatin or not (Figure S7). Consequently the two H2A.Z chaperones, Swr1C and Nap1/CHD may distinguish between free or loaded H2A.Z based on its acetylation status (Figure 7).

### Msc1 action

Msc1 is the largest of the seven JmjC domain proteins in fission yeast and we found it exclusively in Swr1C with no evidence that it occurs in any other complex or as free protein. JmjC domain proteins have raised considerable interest recently because of their ability to demethylate lysines in histone tails [62],[63]. However a thorough bioinformatic analysis of JmjC domains indicated that Msc1 is probably not a demethylase because it lacks key residues in the catalytic domain [64]. Msc1 is a member of the highly conserved Lid/Jarid1 family, which is based on a highly conserved architecture of seven protein domains arrayed in the same N- to C-terminal order (Figure S1). This architecture indicates an integration of several conserved functions in addition to action by the JmjC domain. In addition to Msc1, *S. pombe* has another Lid/Jarid1 member, Lid2, which was found in a complex with subunits of the Set1 H3K4 methyltransferase complex [65] and serves to regulate heterochromatin [66].

Despite much recent activity, it remains unclear how JmjC proteins function to control chromatin. Our proteomic data confines Msc1 function to H2A.Z. Consequently Msc1 presents a good opportunity to understand the action of a JmjC protein.

The finding that the loss of Msc1 leads to ectopic incorporation of H2A.Z into the inner centromeric and subtelomeric chromatin was completely unexpected. None of the known mechanisms for chromatin establishment or maintenance offer an explanation [67]. These mechanisms are all based on cis-acting propagation of chromatin status, which directs the incorporation of new histones whether by RC or RI mechanisms [15]–[18]. To our knowledge, the finding that Msc1 is required to exclude H2A.Z occupancy from two distinct chromatin domains is the first example of a mechanism that appears to prevent the incorporation of a histone variant into the wrong nucleosomes. How Msc1 serves this role remains to be determined but it is notable that neither chromatin domain exists in budding yeast, which also does not contain an Msc1-like subunit in Swr1C. Because H2A.Z incorporation into centromeric or ST chromatin does not require Swr1C, the simplest explanation involves Msc1 directing Swr1C to remove H2A.Z from these domains. However other more complicated explanations are possible. Because Msc1 has been described to be an E3 ubiquitin ligase [68], possibly ubiquitinylation of H2A.Z plays a role in preventing incorporation or facilitating removal from these ectopic sites.

Recent work on another *S. pombe* JmjC/PHD finger protein, Epe1, has identified roles in the maintenance of heterochromatin [69]–[71], although the mechanism remains elusive. It has been suggested that Epe1 is not a demethylase but a hydroxylase (like the original JmjC/cupin domain protein, FIH; [70],[72]). This suggestion was supported by a consideration of conserved and non-conserved amino acids. We note that Msc1 similarly lacks the important signature amino acids for demethylase activity but may retain some characteristics of hydoxylase activity.

Msc1 contains three different but highly conserved PHD fingers [73]. PHD fingers encompass diverse functions [74] but many bind methylated or unmethylated lysines in histone tails [75]–[77]. Hence many PHD fingers serve as ‘readers’ of the post-translational status of nucleosomes. Similarly the JmjC domain, whether active or inactive as a lysine demethylase, also has the potential to read and possibly edit the post-translational status of lysine methylation in nucleosomes. Hence Msc1 is well suited to regulate chromatin status in trans, especially to regulate the RI Swr1C histone chaperone. We therefore suggest that other JmjC proteins, particularly the Lid/Jarid1 family, also serve to ‘read’ chromatin status and thereby convey information to regulatory processes.

### ST-chromatin

H2A.Z is absent from sub-telomeric regions (ca 80kb). The transition from the normal euchromatic H2A.Z pattern to the sub-telomeric region appears to be sharp and coincides with an altered profile of H3K4me2, the presence of retroviral insertions and also presumably the furthest limit of Swi6 binding and H3K9 methylation, which spread from the telomeres [58]. We suggest the term ST-chromatin for this subtelomeric region to distinguish it from the densely H3K9 methylated heterochromatic telomeres and the H3K4me2 euchromatin of the chromosomal arms. In addition to the lack of H2A.Z and uniformly lower levels of H3K4me2, we also note that ST-chromatin is characterized by several distinct features including lower levels of H4K5/K12 acetylation than euchromatin and a higher density of H3 (Table 1). Inner centromeric (IC) chromatin also has uniformly lower levels of H3K4me2 than euchromatin. Hence it is possible that similarities between ST- and IC-chromatin, such as low H3K4me2, account for the similar faulty incorporation of H2A.Z in the absence of Msc1. Notably, forced selection for neocentromere formation, after Cre recombinase centromere deletion, occurred in ST-chromatin [78], and the authors favoured the explanation that the adjacent telomeric heterochromatin influenced the selection of the neocentromeric site. In contrast, we suggest that the similarity between ST- and IC-chromatin is the primary reason. This could be tested by Cre mediated deletion of the centromere on chromosome 3, which has subtelomeric ribosomal repeats rather than ST-chromatin (Figure S4).

Because many meiotic specific genes are found in this domain, it appears that ST-chromatin is an example of regulation of a gene expression program by chromatin domain status. Msc1 is required to maintain this status. In its absence, many genes are derepressed. Notably this derepression extends beyond the ST/euchromatin boundary into euchromatin. Gene derepression in ST-chromatin was not only found in the absence of Msc1 or Swr1, which provoke ectopic H2A.Z deposition into ST-chromatin, but also paradoxically in the absence of H2A.Z, which is normally absent from this domain. This indicates that the maintenance of ST-chromatin requires euchromatic H2A.Z. Furthermore gene repression in ST-chromatin requires Clr3 and Hrp1/3 (Table 1). This evidence provides further reasons to conclude that the genes in ST-chromatin are coordinately regulated by chromatin status.

## Materials and Methods

### Immunoprecipitation

Swr1C purifications from TAP-tagged baits and mass spectrometry identification of complex members were carried out as described elsewhere [47]. H2A.Z purification for MS was carried out according to the standard TAP-tag IP protocol, except bound material was eluted from IgG beads in 0.5M Na-acetate (pH 3.4) and lyophilized to dryness. Samples were reconstituted in HPLC buffer A (5% ACN + 0.1% TFA) and separated by C4 RP-HPLC over a linear acetonitrile gradient. Fractions were collected, lyophilized and digested with Arg-C protease for MS analysis.

### Histone purification

Histones were purified using a protocol adapted from budding yeast [79]. Briefly, harvested yeast pellets from 2L log-phase cultures were homogenized using a beadbeater (BioSpec) in a modified Nuclear Isolation Buffer (0.25M Sucrose, 60mM KCl, 15mM NaCl, 5mM MgCl2, 1mM CaCl2, 20mM HEPES pH8.0, 0.5mM spermine, 2.5mM spermidine, 0.8% Triton X-100, 10mM Na-butyrate and protease inhibitors). The homogenate was centrifuged at 32,000g for 15 minutes, the crude chromatin pellet resuspended in 0.25N HCl, sonicated and rotated at 4°C for one hour. Acid insoluble material was cleared by centrifugation and discarded. Acid soluble material was purified in batch using BioRex70 ion exchange resin (Biorad). Samples were dialyzed against HPLC buffer A and separated by two rounds of RP-HPLC (C4 and C18) over multi-step acetonitrile gradients. Histone-containing fractions from the C4 separation were collected, re-separated over a C18 column, collected again, lyophilized, and digested with Arg-C for MS analysis.

### Mass spectrometry

Arg-C digested samples were first treated with propionic anhydride or deuterated acetic anhydride [80] and directly separated by C18 nanoLC according to standard conditions, and analysed on-line by an LTQ-Orbitrap mass spectrometer (ThermoFinnigan). Survey scans were conducted using the Orbitrap mass analyzer and MS/MS spectra acquired on the linear trap using a standard data dependent acquisition method. Raw data was converted and submitted to MASCOT database searching including lysine methylation, dimethylation, tri-methylation, propionylation and acetylation as variable modifications. Relative quantification of histone peptides was carried out using Xcalibur software (ThermoFinnigan) by extracting the areas of chromatographic peaks of the differentially modified parent ions.

### Chromatin immunoprecipitation and microarrays

ChIP was carried out as previously described [52],[81]. Immunoprecipitated DNA was amplified and hybridized to Affymetrix tiling arrays. Microarrays were carried out in duplicate for both ChIPs and WT input (Affymetrix GeneChip *S. pombe* 1.0FR Arrays) at Pearson correlation coefficients of r>0.97. Probes are tiled for both strands of the genome at an average of 20 base pair resolution. Antibodies used were against H3 (ab1791, Abcam) and H2A.Z-myc (9E10, ab10826, Abcam). Expression arrays were carried out as described [82].

### ChIP-chip data analysis

Raw data from Affymetrix (.CEL format) was analyzed by Affymetrix Tiling Analysis Software (TAS) v1.1 using quantile normalization plus scaling and assigned with a bandwidth of 100. The data was normalized with DNA input and each probe was assigned to the *S. pombe* genome (September edition 2004, Sanger center UK) coordinates in TAS. Visualization of data was performed using the Affymetrix Integrated Genome Browser (IGB). The resulting linear ratio was extracted for each probe position, defined as the center (13th) base coordinate for each 25-nucleotide probe.

Data sets from ChIP on chip experiments were used to map all coding genes onto an average gene, using a similar method as previously described [83],[84]. Briefly, we used the upstream intergenic region and part of coding region for each gene. The analyzed region was −800 to +2800 bp from respectively the start codon of the gene with a 20bp resolution. Values for each probe were attributed to the closest assigned position. Gene expression data was normalized to genomic DNA fragmented by DNAse1 [57] using TAS and the genes were assigned into five expression categories according to their linear signal intensities for sense RNA (arbitrary units; A.U.). In this way, a matrix was generated with 180 columns of 20 bp and a row for each gene in the fission yeast genome. Each column was then averaged vertically for each subgroup of expression to create the average binding values (H3Cter, H2AZ) along each position.

## Supporting Information

Figure S1 Msc1 is a conserved JmjC-domain containing Jarid family member. Multiple amino acid alignment of H. sapiens Jarid1A, Jarid1C, *S. pombe* Lid2 and Msc1 using the colour coding of Gibson et al, TiBS, 19, 349–53 1994. Conserved protein domains are indicated.
(3.77 MB PDF)

Figure S2 Further analyses of euchromatic H2A.Z ChIP. (A) To validate the ChIP-chip, samples were amplified by semi-quantitative PCR using primers to IGR and ORF regions according to the scheme for the gene SPAC12G12.01c, which is also shown in Figure 2B. The “beads” control demonstrates low background binding in an IP carried out without antibody. The “IN” input control was amplified from un-precipitated chromatin and was used to normalize the semi-quantitative PCR. (B) All data from the 20bp tiling arrays were ordered with respect to the initiating methionine of each gene and binned into IGR (intergenic region) or ORF (open reading frames). Then Log2(IGR/ORF) ratios were calculated for each gene and then binned for the histogram. The plot shows that H2A.Z was enriched in IGRs as opposed to open reading frames, and this enrichment is dependent on Swr1 but is largely independent of Msc1. (C) Moving average plots were generated to compare H2A.Z and H4K5, K12 and K16 acetylation genome-wide distributions at IGRs using data from Wiren et al, EMBO J 24, 2906–18 2005. (D) As in (C), for H3K9 and H3K14 acetylation at IGRs. (E) Venn diagrams showing the overlap between IGRs enriched in H2A.Z and H4K16 acetylation in WT, msc1Δ, and swr1Δ. (F) As in (C), but comparing H2A.Z distribution in WT, msc1Δ, and swr1Δ vs H4K16 acetylation data at IGRs. (G) As in (F) for H3K14 acetylation.
(1.35 MB EPS)

Figure S3 The inner centromeres of Chromosomes 2 and 3 also acquire H2A.Z and increased H3 levels in the absence of Swr1 or Msc1. (A) Chromosome 2 H2A.Z-myc ChIP. (B) The corresponding H3 ChIP. (C) Chromosome 3 H2A.Z-myc ChIP. (D) The corresponding H3 ChIP. The structural features are labeled below the panels.
(4.97 MB EPS)

Figure S4 H2A.Z ChIP on the sub-telomeres of all three chromosomes. ChIP-chip binding profiles for H2A.Z-myc at 180kb regions at both ends of chromosome 1 (A, B), 2 (C, D), and 3 (E, F) in WT, msc1Δ, and swr1Δ. Open reading frames are represented by black boxes and LTR retrotransposon elements by orange boxes. The dotted lines demonstrate the approximate transition points between chromatin domains. The ribosomal gene repeats lie at the far left (E) and far right (F) of chromosome 3 and are represented by the large black boxes. The probe distribution in these regions is very sparse.
(5.05 MB EPS)

Figure S5 Purification of H2A.Z-TAP and H2A.Z-associated H2B for MS. (A) Coomassie stained SDS-PAGE gel and dot blot of fractions from the C4 RP-HPLC separation of H2A.Z-TAP in WT. The dot blot was probed with an antibody directed against the TAP-tag. Histones are indicated. (B) Chromatogram of RPHPLC separation (absorbance 214nm) in WT, msc1Δ, and swr1Δ.
(5.95 MB EPS)

Figure S6 H2A.Z is found in two isoforms, distinguished by two N-terminal methionines, and can be acetylated on all four lysines of the N-terminal tail. (A) Listed are H2A.Z peptides as detected by LC-MS/MS analysis. The observed and predicted masses are presented, and the difference between these values (delta) given in parts per million (ppm), as are the number of missed cleavages (from an Arg-C digest), the amino acid sequence, the detected modifications and Mascot scores for MSMS fragmentation spectra. Note that each acetylation isoform was detected as 2+ and 3+ charge states. (B) MSMS spectra for H2A.Z 1-22ac4 and (C) H2A.Z 3-22ac4 peptides (parent ions not shown). Fragmentation is also represented schematically.
(0.82 MB EPS)

Figure S7 Acetylation of H2A.Z-associated H2B is not strongly affected by the loss of either Swr1 or Msc1. Relative quantification of H2A.Z-associated H2B 1–18 acetylation from WT, msc1Δ, and swr1Δ, plus global H2B 1–18 acetylation levels in WT. H2B can be acetylated on three lysine residues of the N-terminal tail (K5, K10 and K15) and the N-terminus, and is predominantly found acetylated at all three potential sites (termed the 3ac isoform) in WT whether associated with H2A.Z or not. The number of acetyl marks are indicated either as acetylation of the N-terminus itself (“Nt Ac”) or of tail lysine residues (“Ac”).
(0.39 MB EPS)
