# Supplementary figures and images for: The Schizosaccharomyces pombe JmjC-Protein, Msc1, Prevents H2A.Z Localization in Centromeric and Subtelomeric Chromatin Domains
Source: PLoS Genet. 2009 Nov 13;5(11):e1000726. doi: 10.1371/journal.pgen.1000726 (PMC2770259; doi:10.1371/journal.pgen.1000726)

# Buchanan et al.

## Supplementary Figure 1

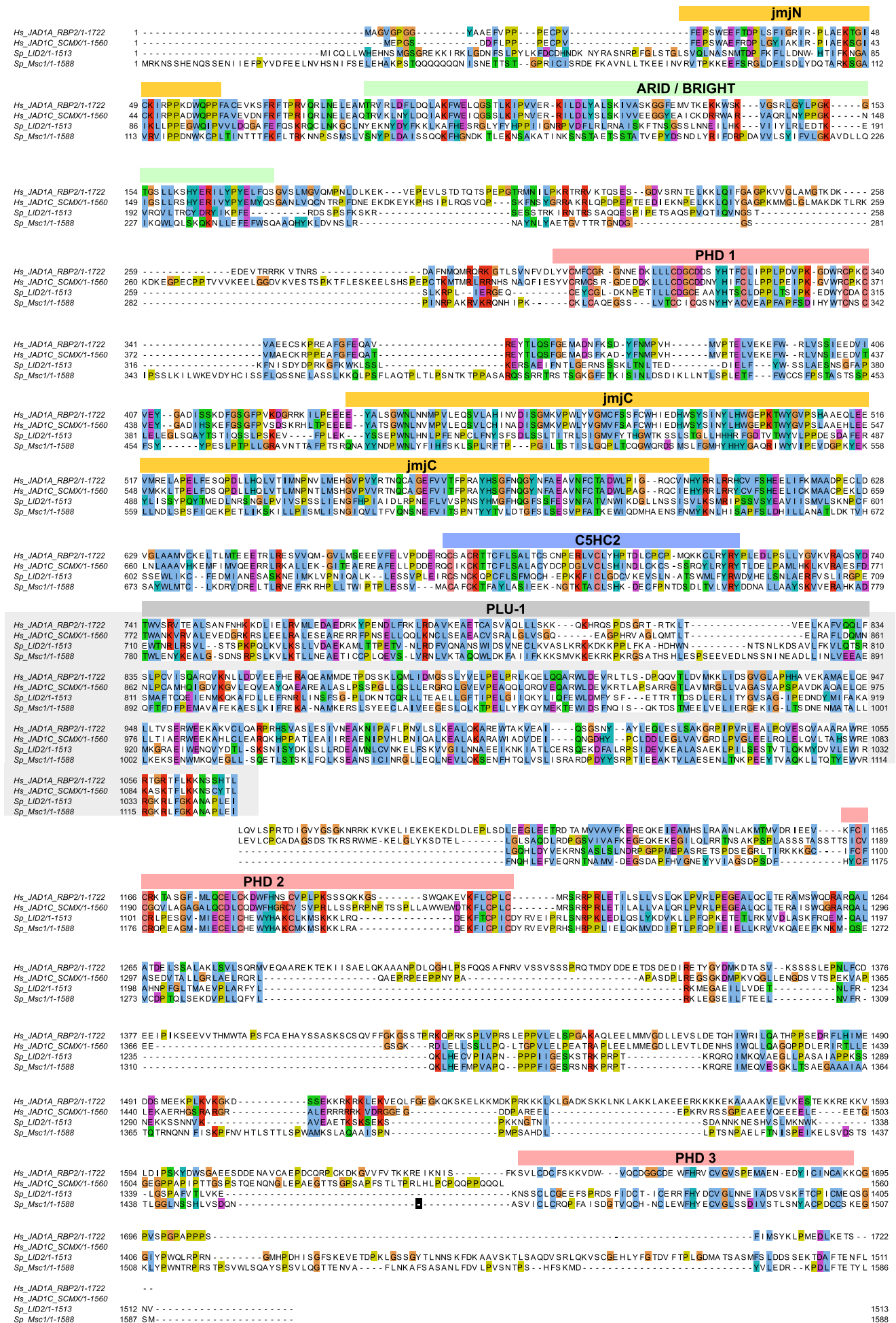

Supplement: Figure S1 — Msc1 is a conserved JmjC-domain containing Jarid family member. Multiple amino acid alignment of H. sapiens Jarid1A, Jarid1C, S. pombe Lid2 and Msc1 using the colour coding of Gibson et al, TiBS, 19, 349–53 1994. Conserved protein domains are indicated. (3.77 MB PDF) [file pgen.1000726.s001.pdf]
